# Monitoring Hip Joint Muscle Function in Osteoarthritis Patients Following Arthroplasty: A Prospective Cohort Study

**DOI:** 10.3390/jcm14030976

**Published:** 2025-02-03

**Authors:** Dorota Staniak, Alicja Wójcik-Załuska, Krzysztof Sokołowski, Małgorzata Drelich, Izabela Świetlicka, Monika Prendecka-Wróbel, Teresa Małecka-Massalska

**Affiliations:** 1 Department of Clinical Physiotherapy, Medical University of Lublin, 20-059 Lublin, Poland; alicja.wojcik-zaluska@umlub.pl; 2 Department of Physiotherapy, Faculty of Health Sciences, Vincent Pol University, 20-816 Lublin, Poland; sokolowskik@interia.pl; 3 Department of Rehabilitation, Medical University of Lublin, 20-059 Lublin, Poland; malgorzata.drelich@umlub.pl; 4 Department of Biophysics, University of Life Science, 20-950 Lublin, Poland; izabela.swietlicka@up.lublin.pl; 5 Physiology Department, Medical University of Lublin, 20-059 Lublin, Poland; monika.prendecka-wrobel@umlub.pl (M.P.-W.); teresa.malecka-massalska@umlub.pl (T.M.-M.)

**Keywords:** hip osteoarthritis, arthroplasty, MyotonPro, muscle function

## Abstract

**Background/Objectives**: Osteoarthritis (OA) is a chronic and progressive joint disease, leading to functional limitations and significantly impairing the quality of life. Muscle weakness, reduced mobility, and compensatory biomechanical changes are common consequences, further exacerbating functional decline. The aim of this study was to assess the impact of hip osteoarthritis on muscle functionality and to evaluate the effectiveness of hip arthroplasty using the MyotonPro device to measure key biomechanical parameters, i.e., tension, stiffness, and flexibility. **Methods**: This cohort study included 40 patients (17 women and 23 men; mean age 64.55 ± 10.49 years) with advanced hip OA (Kellgren–Lawrence grade III–IV) undergoing hip arthroplasty. Measurements of muscle tension (F), stiffness (S), and flexibility (D) in the gluteus maximus, rectus femoris, and biceps femoris were performed at three time points: before surgery, on postoperative days 8–10, and one month after hospital discharge. Pain (VAS), balance (Tinetti scale), and functional ability (WOMAC index) were also assessed. **Results**: Hip arthroplasty significantly reduced pain levels (VAS: 6.38 ± 0.28 preoperatively to 1.88 ± 0.22 postoperatively, *p* < 0.001) and improved functional ability (WOMAC: *p* < 0.001). Muscle tension and stiffness of the gluteus maximus initially increased after surgery (tension: 11.57 ± 0.32 to 12.15 ± 0.38, *p* = 0.009), reflecting compensatory stabilization but decreased by the final evaluation. Flexibility improved significantly over time (*p* = 0.014). The biceps femoris muscle exhibited a significant reduction in tension one month postoperatively (*p* = 0.015), alongside decreased stiffness (*p* = 0.015) and enhanced flexibility. The rectus femoris muscle showed minor changes in biomechanical properties, with no statistically significant differences detected. **Conclusions**: Osteoarthritis significantly impacts muscle function, reducing the gluteus muscle tension and stiffness, which compromises joint stability and triggers compensatory activity in the rectus femoris and biceps femoris muscles. Postoperative rehabilitation is essential for improving flexibility and addressing compensatory muscle tension.

## 1. Introduction

Osteoarthritis (OA) is the most common joint disease in the world. It is a chronic disease manifested by an imbalance between the processes of degeneration and regeneration of articular cartilage. It is also accompanied by other changes within the entire joint, leading to a limitation of its functions. Primary and secondary forms of osteoarthritis have been classified. The former is idiopathic, while the secondary form is the result of factors damaging the joint surfaces [1,2,3].

The occurrence of this disease becomes more and more common with age, and its consequences lead to a significant deterioration of the quality of patient’s life. Approximately 4% of the global population experiences symptoms of osteoarthritis in the knee and hip joints; however, the actual number of people affected by this condition is significantly higher. Radiological changes in the hip joints, along with osteoarthritis symptoms, are observed in about 10% of the global population. The occurrence of hip joint osteoarthritis is common, with the highest prevalence recorded in Europe, where it reaches 12.6%. It may be suspected that factors such as gender, age, increased body weight, or genetic predisposition increase the possibility of developing OA. Osteoarthritis significantly limits the patient’s independence, gradually reducing mobility [4,5,6,7,8,9].

Pain in the affected joints is a major symptom of OA, restricting patients from physical activity, while prolonged immobility affects muscle condition. Another observed symptom is stiffness that increases after a period of immobility. The reduced range of joint mobility effectively makes it difficult to perform everyday activities [5,6].

In patients with osteoarthritis of the lower limb joints, usually affecting the hip area, muscle weakness is often observed. Hip abductors, such as the gluteus medius muscle, are essential for postural stability and dynamic balance. The weakness of these muscles leads to pelvic instability, which manifests as the Trendelenburg sign, where the pelvis drops on the side opposite the stance leg while walking. This instability reduces the efficiency of the gait pattern, which in turn leads to compensatory biomechanical changes, placing additional strain on the other structures of the lower limbs, such as the knees or ankles. Increased stiffness of muscles, such as the gluteal muscles, rectus femoris or tensor fasciae latae, contributes to a reduced range of motion in the hip joint, which eventually impairs the ability to perform functional tasks, like walking, climbing stairs, and even standing up from a sitting position. As a result, patients often adopt a compensatory posture, causing excessive strain on other muscles and joints, increasing the risk of overuse and injury. Prolonged avoidance of functional loading, due to pain and discomfort associated with osteoarthritis, also contributes to muscle atrophy. This is particularly evident in anti-gravity muscles, such as the quadriceps femoris, which plays a key role in stabilizing the knee joint during standing and walking [10,11,12,13].

**Figure 1:**
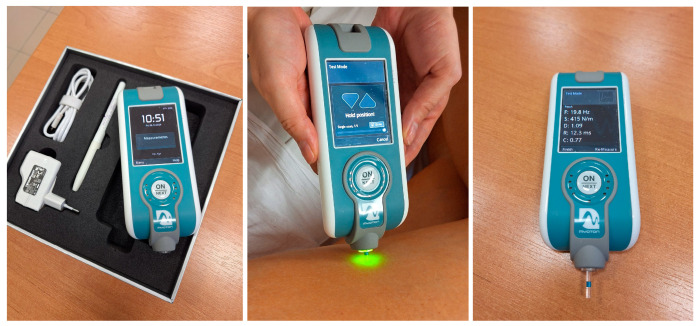
MyotonPro device. Measurement involves examining the mechanical properties of muscles using short impulse and mechanical force, generating damped muscle vibrations. The built-in accelerometer records the oscillatory response of the muscle and translates it into records like tension (F), stiffness (S), and flexibility (D). The photo was taken in the Department of Orthopedics and Traumatology of the University Clinical Hospital No. 4 in Lublin.

This study attempted to assess the changes occurring in the muscles around the hip joint as a result of osteoarthritis and the impact of hip replacement surgery on their function. MyotonPro (Myoton AS, Talinn, Estopnia) is a portable diagnostic device used to assess the biomechanical properties of soft tissues, such as muscles, tendons, ligaments, and skin. The device works by generating short mechanical impulses that are applied to the skin’s surface and then recording the tissue’s response to these impulses. Based on the data collected, MyotonPro allows the assessment of parameters like stiffness, elasticity, tone, viscosity, and muscle relaxation time. In studies of patients with osteoarthritis, MyotonPro can be used to assess altered muscle properties resulting from joint pathology. Muscle stiffness and tone are key parameters that may change in patients with OA, particularly around the hip, knee, and ankle joints. Assessing these parameters allows for objective monitoring of the condition of muscles and soft tissues at different stages of treatment, both surgical and non-operative. By using MyotonPro, it is possible to obtain objective, repeatable results regarding the condition of soft tissues, contributing to a better functional assessment of patients and enabling more precise adjustment of therapy (see Figure 1). This device can also help monitor the effects of treatment and rehabilitation progress, thus, supporting patients more effectively in restoring physical fitness and improving quality of life [14,15,16].

The primary aim of this study was to evaluate the functional impact of advanced hip osteoarthritis on key biomechanical properties—muscle tension (F), stiffness (S), and flexibility (D)–of the hip joint muscles and to compare these parameters before and after hip arthroplasty. The study hypothesized that osteoarthritis significantly alters the biomechanical properties of the gluteus maximus, rectus femoris, and biceps femoris muscles and that hip arthroplasty restores these properties by reducing compensatory muscle activity while improving flexibility and stability over time. Additionally, the study aimed to highlight the unique application of the MyotonPro device in objectively quantifying muscle biomechanics during the preoperative period, providing a novel perspective on postoperative recovery dynamics.

## 2. Materials and Methods

### 2.1. Study Design

**Figure 2:**
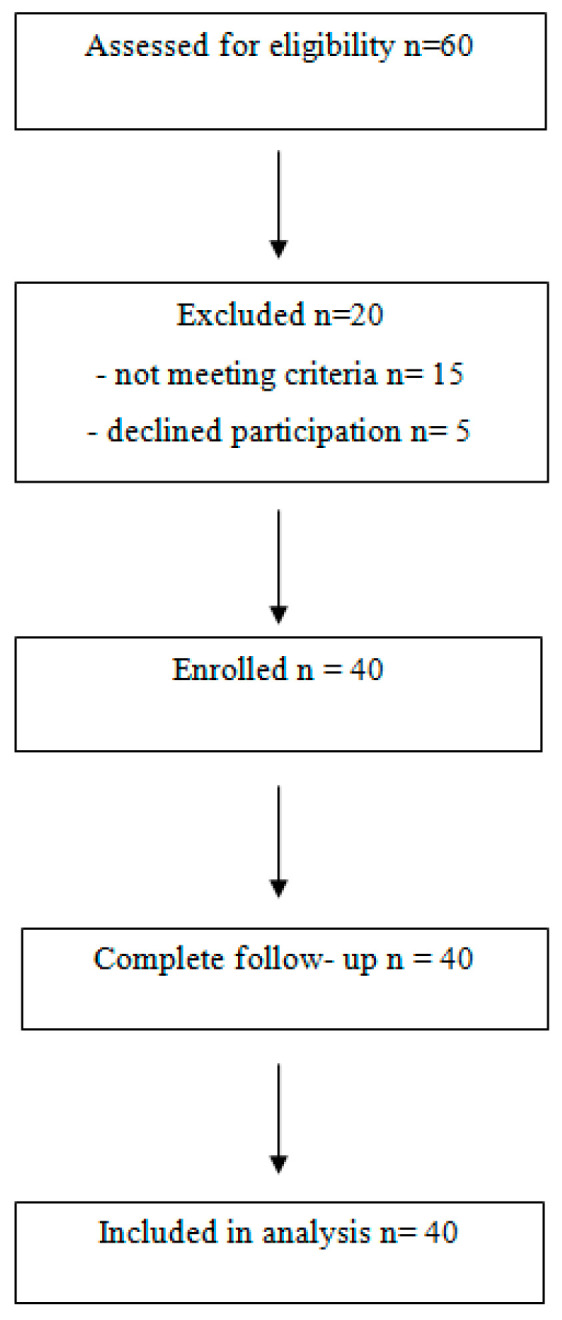
A flow diagram presenting the number of patients at each stage of the research.

The study was conducted in the Department of Orthopedics and Rehabilitation of the Independent Public Clinical Hospital No. 4 in Lublin, Poland from 2021 to 2023. Recruitment of participants occurred between January 2021 and December 2022, with follow up examinations completed by June 2023. The flow diagram showing the number of participants at each stage of the study is describe at Figure 2. The study was voluntary, and all participants were informed in detail about the purpose, methodology, and scope of the study. Written informed consent was obtained prior to inclusion. The research was approved by the bioethics committee of the Medical University of Lublin, Poland—No. KE-0254/149/2021 (24 June 2021).

### 2.2. Study Population

The study was conducted among patients with advanced osteoarthritis of the hip joint (categorized as Kellgren–Lawrence grade III–IV) who underwent arthroplasty. The group of 40 patients with hip osteoarthritis qualified for the study included 17 women and 23 men. The average age of respondents was 64.55 (±10.49) years and the average weight was 85.15 (±15.49) kg. Recruitment involved reviewing patient records at the orthopedic department and identifying those meeting the inclusion criteria. Eligible individuals were invited to participate during pre-surgical consultations, where they were informed about the study objectives and procedures. Major inclusion criteria were pain and advanced osteoarthritis of the hip joint. The exclusion criteria were the history of stroke; cerebrospinal injuries; previous lower limb amputations; multiple sclerosis; rheumatoid arthritis; active cancer; and early or severe postoperative complications. The analyzed group of patients was balanced regarding key demographic and clinical characteristics to minimize the impact of potential confounders such as sex, age, BMI, activity, and pain level. It should be underlined that participants were recruited based on clearly defined inclusion and exclusion criteria to minimize selection bias. This ensured a homogenous and representative cohort of patients undergoing hip arthroplasty.

Patients qualified for the study underwent surgery under spinal anesthesia. During surgery, patients were placed on the opposite side and posterolateral access to the joint was performed. Each patient received fluid therapy at a dose of 10 to 15 mL per kg of body weight during surgery and 1000 to 1500 mL of crystalloids during the first postoperative period. Analgesic treatment was provided for two days after the procedure in all patients. After surgery, patients received rehabilitation programs in a hospital unit. Patients were activated the second day after surgery. Postoperatively, antithrombotic, isometric, and active exercise were used together with verticalization and gait re-education.

### 2.3. Patients’ Evaluation

All patients were evaluated at three time points. The first evaluation was conducted prior to hip arthroplasty surgery, on the day of hospital admission. The second evaluation took place on the 8–10th postoperative day, following the rehabilitation process. The final evaluation was conducted one month after discharge from the hospital. To reduce information bias, data were collected using validated tools, and all measurements were performed to ensure objectivity and reproducibility. Data collection protocols were standardized across all three time points to maintain consistency.

To collect basic information about patients’, an author’s questionnaire was used. It included questions about socio-demographic situations, history of osteoarthritis, previous treatment of OA, associated diseases, severity of pain, and the impact of OA on functioning and performance of daily activities.

To assess the intensity of pain, a visual analog scale (VAS) was used. A Tinneti scale and WOMAC (Western Ontario and McMaster Universities Osteoarthritis Index) was used to evaluate patients’ balance, quality of gait, and the ability to independently perform simple tasks of everyday activities.

To assess changes in the muscle tone, stiffness and elasticity caused by osteoarthritis as well as differences in the properties of muscles around the hip joint after arthroplasty was performed by a MyotonPro device (Myoton AS, Tallinn, Estonia).

### 2.4. MyotonPro Measurements

MyotonPro is a device that enables non-invasive examination of the mechanical properties of muscles using a short pulse and mechanical force that generates damped muscle vibrations. The built-in accelerometer records the oscillatory response of the muscle, making it possible to examine parameters such as muscle tone- which is natural oscillation frequency (Hz), dynamic stiffness (characterizing soft tissue resistance for a deformation (N/m)), and flexibility (characterizing elasticity).

**Figure 3:**
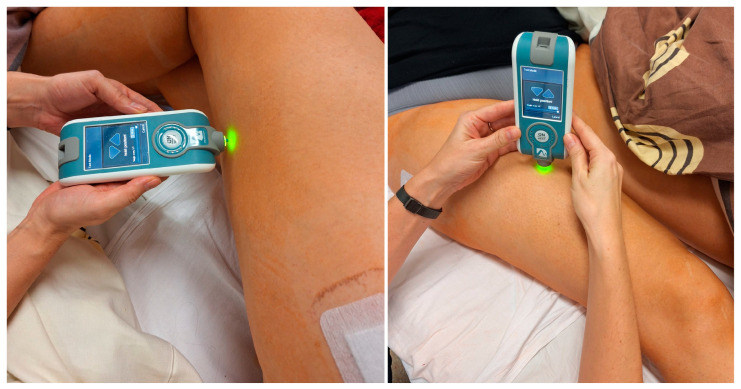
Measurement of tension, stiffness, and flexibility by the MyotonPro device. Measurement was taken in a lying position, and the device was placed perpendicular to the tested muscle. The photo was taken in the Department of Orthopedics and Traumatology of the University Clinical Hospital No. 4 in Lublin.

In our study, we measured parameters for three muscles of lower limb that play a key role in stabilizing and functioning of the hip joint, that is the gluteus maximus, rectus femoris, and biceps femoris. The measurement was performed in a lying position, knees extended, and hips in a neutral position. The MyotonPro device was placed perpendicular to the tested muscle and halfway along its length (see Figure 3). The measurement place was labeled with a marker on the skin. All patients rested for 5 min before measurement and were asked to relax their muscles.

### 2.5. Statistical Analysis

The collected data were analyzed in terms of their nature. Categorical variables were characterized by the structure indices, namely the absolute counts and percentage shares (%). The normal distribution (Shapiro–Wilk test) and variance homogeneity (Levene’s test) were checked for the continuous variables that were presented as means with standard errors (SE). There were no missing data in the dataset, as all patients completed the study and provided complete data at all three time points. Therefore, no imputation or alternative handling methods were required.

Measured variables changes in time were analyzed using repeated measures ANOVA. In cases where the assumptions of the analysis were violated, the nonparametric equivalent, namely Friedman ANOVA, was applied. When significant differences were identified across the analyzed time points, post hoc analysis was conducted to determine which specific time points differed significantly. Post hoc tests were adjusted for multiple comparisons to control for Type I error, ensuring the robustness of the statistical conclusions. Differences in tension (F), stiffness (S), and flexibility (D) between the healthy and affected limb at a given time point were analyzed using the Mann–Whitney U test. Potential confounders, such as age, sex, BMI, and baseline pain and activity levels, were identified based on clinical relevance and existing literature. Statistical tests (Student’s *t*-test, Pearson correlation, or Spearman’s correlation) were used to check differences between subgroups and their potential influence on the outcomes.

All the statistical evaluations were performed using a two-sided approach, with a significance threshold of α = 0.05. The statistical assessments were conducted using RStudio (1 September 2023, build 494, Integrated Development for R. RStudio, PBC, Boston, MA, USA; URL: http://www.rstudio.com/), STATISTICA 13.0 (TIBCO Software, Palo Alto, CA, USA), and OriginPro 2022b v.9.95 (OriginLab, Northampton, MA, USA) software.

## 3. Results

### 3.1. Characteristics of the Research Group

In total, 40 individuals were recruited for the study, including 17 women and 23 men. The participants’ ages ranged from 54.04 to 75.04 years. Their heights varied between 161.79 cm and 178.41 cm and weight ranged from 69.66 kg to 100.65 kg with males being significantly higher and heavier than females. Despite the variations, the BMI of patients remained stable regardless of sex (Table 1).

The relationships between potential confounders and measured parameters, assessed using correlation analysis (Pearson or Spearman’s), showed almost no significant (at *p* < 0.05) associations between tension, stiffness, and flexibility at any time stage and sex, age, BMI, or the initial levels of VAS, WOMAC, and Tinetti scales (Tables S1–S3) for both operated and not operated limb. Only a few correlation coefficients turned out to be significant; however, all of them were negligible to weak.

The assessment of pain level, balance and functional ability was conducted for all participants. The pain level, measured by the VAS, decreased significantly (*p* < 0.001) from 6.38 (±0.28) to 1.88 (±0.22) during the examination one month after the surgical procedure. Functional ability, assessed using the WOMAC scale, showed a statistically significant increase at the last stage of the examination. Similarly, one month after surgery, patients’ balance (measured with the Tinetti scale) returned to its initial preoperative level, following a significant decline observed 8–10 days post surgery (Table 2).

### 3.2. Myoton Measurements

Measurements of tension (F), stiffness (S), and flexibility (D) of the gluteus maximus, rectus femoris, and biceps femoris were performed by the MyotonPro device. The measurements were performed for the operated and non–operated sides at three time intervals: before surgery, on the 8–10th day after surgery, and one month after patient’s discharge from hospital.

#### 3.2.1. Tension, Stiffness, and Flexibility of the Gluteus Maximus Muscle

The tension (F) of the gluteus maximus before the operations on both sides was at a comparable level, without significant statistical differences (*p* = 0.969), after the operation it increased on the operated side (from 11.57 ± 0.32 to 12.15 ± 0.38) which was statistically significant (*p* = 0.009). At the last stage of the examination, the tension on the operated side decreased, although it remained at a higher level than on the other side- the differences were not statistically significant (*p* = 0.179).

Before therapy, the stiffness (S) of the gluteus maximus muscle was lower on the operated side, although the differences were not statistically significant (*p* = 0.355). After hip arthroplasty, muscle stiffness increased significantly (from 215.1 ± 5.7 to 232.2 ± 12.8), although these changes were not significant (*p* = 0.088) compared to the non-operated side. At the last stage of the examination, muscle stiffness returned to the preoperative level and was comparable to that of the non-operated side.

The elasticity (D) of the gluteus maximus muscle before surgery remained at a similar level on both sides, but after surgery the values increased for both the operated and non-operated side. At the last stage of the study, the flexibility on the operated sides was higher, which was a statistically significant difference (*p* = 0.014).

**Figure 4:**
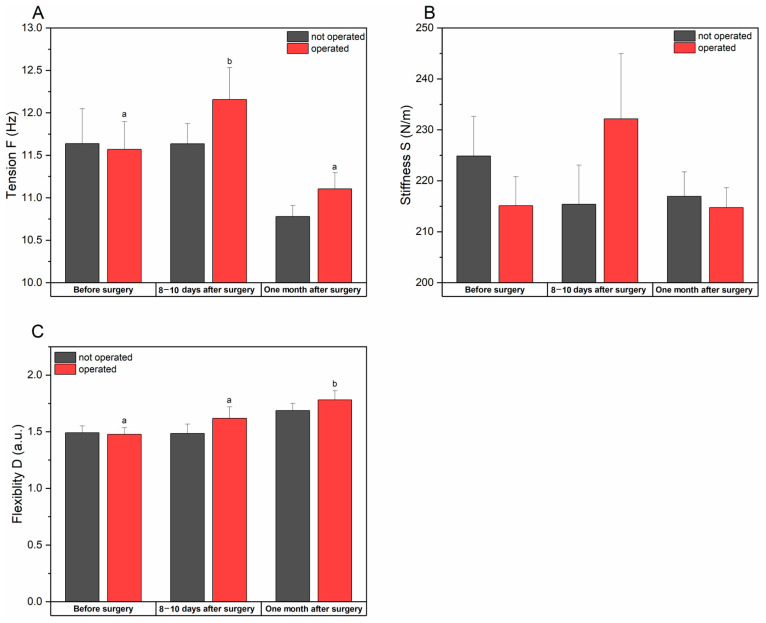
Changes in tension (**A**), stiffness (**B**), and flexibility (**C**) of the gluteus maximus muscle for operated and not operated patients measured at different time points. Data are presented as means ± SE. a and b indicate statistically different measurements between time points at *p* < 0.05.

The analysis of the results show an increase in muscle tension and stiffness after hip arthroplasty, which may indicate compensation for stabilization of the hip joint. Improved flexibility at the last stage of the examination proves the positive impact of rehabilitation, which results in greater mobility of the hip joint (Table 3 and Figure 4).

#### 3.2.2. Tension, Stiffness, and Flexibility of the Rectus Femoris Muscle

The tension (F) of the rectus femoris muscle on the operated side was higher than the non-operated one, but these differences remained statistically insignificant (*p* = 0.534). After the procedure, the tension on the operated side increased significantly compared to the non-operated side (*p* = 0.049), while at the last stage of the study, the tension on the operated side decreased. However, the differences between the sides were not statistically significant (*p* = 0.161).

The rectus femoris muscle stiffness (S) before and after surgery was higher on the operated side, although the differences compared to the non-operated side were not statistically significant. At the last examination, muscle stiffness on the operated side decreased but remained significantly different from that on the opposite side (*p* = 0.292).

The elasticity (D) of the rectus femoris muscle on the operated side was higher than the values for the non-operated one in all the examined time points. However, the differences were not statistically significant. Additionally, no significant changes over time were detected for this parameter for individual sites.

**Figure 5:**
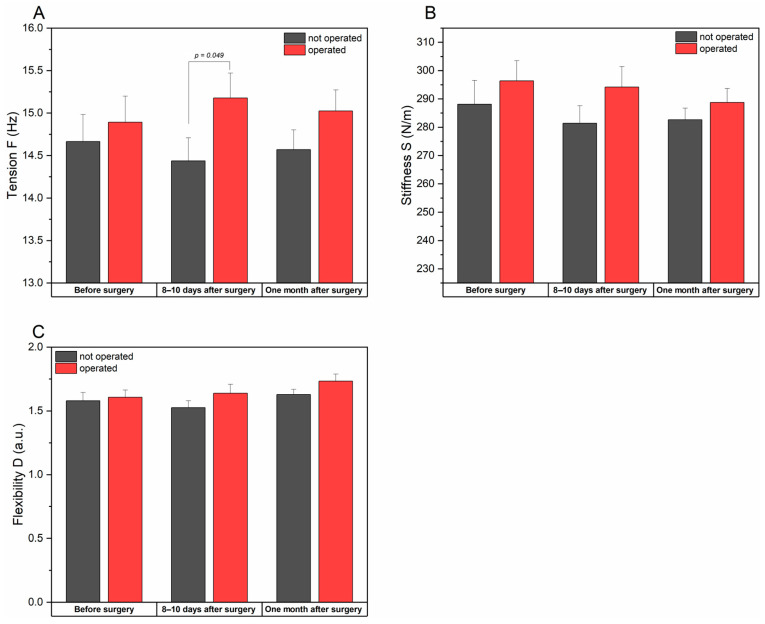
Changes in rectus femoris muscle tension (**A**), stiffness (**B**),and flexibility (**C**) for operated and not operated patients measured at different time points. Data are presented as means ± SE. *p*-value indicates statistically different measurements between sides at *p* < 0.05.

Increased tension of the rectus femoris muscle after surgery may be related to increased compensatory activity when stabilizing the hip joint. More minor changes in stiffness suggest that surgery will not significantly affect the stiffness of the rectus femoris muscle (Table 4 and Figure 5).

#### 3.2.3. Tension, Stiffness, and Flexibility of the Biceps Femoris Muscle

Before surgery, the tension (F) of the biceps femoris muscle on the operated side was significantly higher than on the non-operated side (*p* = 0.035). After the procedure, the tension increased on both sides, while one month after the surgery, the tension on the operated side decreased and became significantly lower than on the opposite side (*p* = 0.049). Tension changes in time for the operated leg were observed, with a significant decrease in the month after the surgery concerning the previous time points (*p* = 0.015).

The stiffness (S) of the biceps femoris muscle before and after the surgery was higher on the operated side, but the differences were not statistically significant. However, at the last examination, muscle stiffness on the operated side decreased significantly (*p* = 0.015) and was notably lower (*p* = 0.035) than the value observed for the not operated side.

Before the procedure, the flexibility (D) of the biceps femoris on the surgical side was lower compared to the contralateral side, although the difference was not significant (*p* = 0.207). After surgery, elasticity increased and exceeded the values of the non-operated side, but even here, the differences were not significant (*p* = 0.132). The flexibility on the operated side remained higher than before the surgery, but the differences between the sides were not statistically significant.

**Figure 6:**
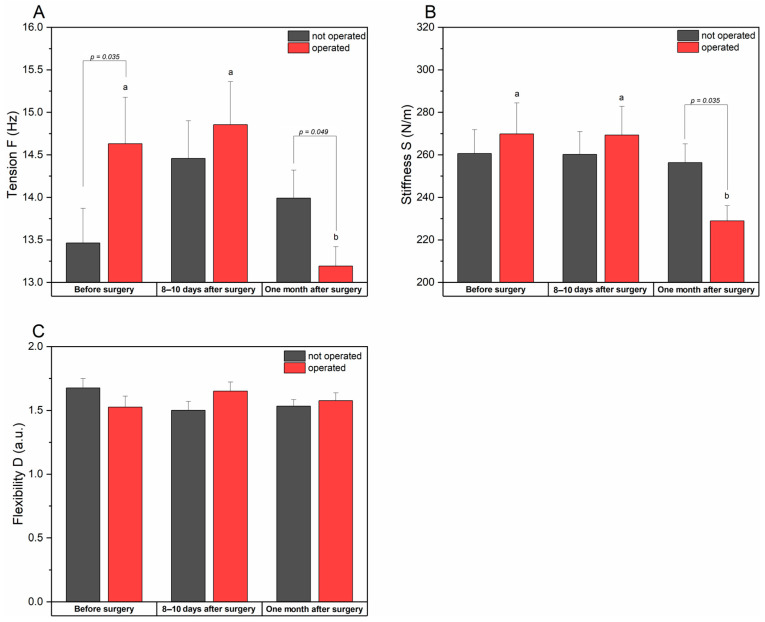
Changes in biceps femoris muscle tension (**A**), stiffness (**B**), and flexibility (**C**) for operated and not operated patients measured at different time points. Data are presented as means ± SE. *p*-value indicates statistically different measurements between sides at *p* < 0.05; a and b indicate statistically different values between time points at *p* < 0.05.

Higher muscle tension and stiffness on the operated side before the surgery may indicate increased compensatory activity. After surgery, function normalization is observed, manifested by reduced tension and stiffness and improved flexibility (Table 5 and Figure 6).

**Figure 7:**
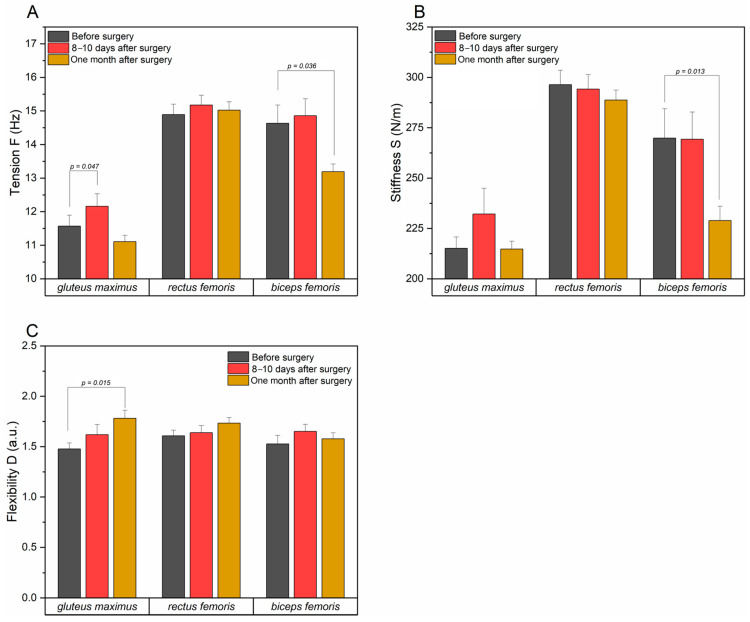
Comparison of muscle properties (tension (**A**), stiffness (**B**), and flexibility (**C**)) for the operated side for gluteus maximus, rectus femoris, and biceps femoris muscles between time points: before surgery, 8–10 days after surgery, and month after surgery.

An increase in the tension (F) of the gluteus maximus muscle in the postoperative examination compared to the preoperative examination may indicate increased stabilization activity of the hip joint. From the analysis of the obtained values of the tested parameters before and one month after the surgery, it can be concluded that the tension (F) returns to normal after some time, which may be the result of rehabilitation. There were no significant changes in stiffness (S) for the preoperative and postoperative examinations, suggesting that the surgery did not significantly affect the biomechanical properties of the muscle. The lack of a significant pre- and post-treatment difference in flexibility (D) suggests that flexibility does not change significantly in the short term after treatment. A meaningful improvement in flexibility at the last stage of the examination compared to the value measured before the surgery indicates a gradual recovery of muscle functionality (Figure 7).

The examination of the tension (F) of the rectus femoris muscle indicated that there were no significant changes between the tested values before and after the surgery and between the examination before and one month after the procedure, which suggests that the muscle tension did not change much. Similar results were obtained for muscle stiffness (S) and elasticity (D), suggesting that these parameters remain stable at each stage of the study (Figure 7).

Results obtained in the examination of the biceps femoris muscle in terms of tension (F) indicated no significant changes in the values of examined parameters before and after surgery, but there was a significant difference between the data obtained before surgery and one month after, which indicates that the tension decreased at the last stage of the examination (0.036). In the case of the stiffness parameter, no significant changes were observed when comparing the values obtained before and after the surgery, which may indicate that the surgery itself had no direct effect on muscle stiffness. There was also a difference between the results of the first and last examination (*p* = 0.013), which may suggest that after a longer period there was a significant reduction in stiffness, which is most likely the result of improved function and better mobility. In terms of muscle elasticity (D), no significant changes were noted, both when comparing the results of examination before and after the procedure, as well as in case of parameters obtained one month after the procedure to the state before the operation, pointing that the flexibility remains stable at every stage of the examination (Figure 7).

For the gluteus maximus and hamstring muscles, a significant improvement in muscle function can be seen after a month, which indicates the effectiveness of rehabilitation after hip joint surgery. The rectus femoris muscle appears to be less involved in the adaptation process, which may mean that its role in compensation is not essential.

## 4. Discussion

Osteoarthritis (OA) of the lower limb joints remains one of the leading causes of impaired mobility and disability. Beyond typical symptoms such as joint pain and stiffness, changes in the structure and function of the surrounding muscles play a crucial role in reducing patients’ physical performance. In hip osteoarthritis, muscle weakness—particularly in the gluteal and abductor muscles—significantly impacts gait quality and the ability to perform daily activities. Our study aimed to investigate the biomechanical changes in the muscle parameters following hip arthroplasty. The results demonstrated notable improvements in muscle tension and stiffness after arthroplasty, particularly in the gluteus maximus and biceps femoris, which play crucial roles in postoperative adaptation. The changes recorded for the rectus femoris muscle were less pronounced. Dias et al., 2022 [11] emphasized the impact of weakened hip stabilizing muscles, such as the gluteal muscles and the tensor fasciae latae, on compensatory load transfer to adjacent structures, including the knee joint. Consistent with these findings, our results showed significant postoperative changes in the gluteus maximus and biceps femoris. These muscles play a key role in postoperative adaptation, highlighting their importance in rehabilitation. In contrast, the smaller changes observed in the rectus femoris align with its limited role in stabilizing the hip joint, as noted in prior research.

In the present work we show normalization of muscle function one month after hip arthroplasty, aligning with the findings of Ismailidis et al., 2021 [17], who reported improved activity in the hip abductor muscles post-surgery. However, in the study of Ismailidis team, it was also shown that, in the long term, muscle strength on the operated side remains lower than on the healthy side, indicating the need for continued rehabilitation to achieve full functional symmetry.

The use of myotonometry in our study provided objective and precise assessment of muscle tension and stiffness, supporting its value as a diagnostic tool. As confirmed by Lettner et al., 2024 [14] and Lee et al., 2021 [18], the MyotonPro device provides precise analysis of the biomechanical properties of muscles in both healthy populations and patients with musculoskeletal disorders. Similar findings were observed by Li et al., 2020 [19]. The authors examined passive stiffness of gastrocnemuis muscle among healthy individuals. As a result, they stated that MyotonPro is a reliable device to measure changes in muscle properties. Our study demonstrated the most significant changes in the tension and stiffness of the gluteus maximus and biceps femoris, consistent with their key roles in hip stabilization and postoperative adaptation.

Chang et al., 2022 [20] and Li et al., 2024 [21] observed increased stiffness in the rectus femoris and other quadriceps components in knee OA, which contributed to impaired gait patterns and increased pain. In contrast, our study found smaller changes in the tension and stiffness of the rectus femoris, which could be attributed to differences in the biomechanics of the hip and knee joints. The rectus femoris has a different role in stabilizing the hip joint, which may explain these discrepancies. Shen et al. (2024) [10] also noted that changes in quadriceps muscle properties significantly affect gait quality and compensatory mechanisms, particularly in knee OA. The comparison between our findings and those focused on knee OA highlights the significant impact of lower limb muscle function on the development and progression of osteoarthritis. The inconsistencies between findings may also be related to the heterogeneity of the studied populations. Chang [20], Shen [10], and Li [21] analyzed muscle changes in patients with knee OA, while our study focused on patients with hip OA. This highlights the need for further research into muscle mechanics in hip OA to better understand their role in treatment and rehabilitation processes.

Despite the strength of our study, several limitations warrant consideration. The relatively small sample size may limit the generalizability of our findings, and the short follow-up period restricts insights into long-term outcomes. Future studies should aim to include larger, more divers cohorts and explore longer-term effects to validate and expand upon our results.

Despite these limitations, our results have practical implications for broader populations. In the study by Martinez-Pozas et al., 2023 [22], in contrast to our results, the application of orthopedic manual therapy did not result in a significant reduction in pain experienced by patients with musculoskeletal disorders. Therefore, as indicated by the findings of our research it is essential to underscore the importance of individualized rehabilitation approaches that account for specific changes in muscle tension and stiffness. By focusing on key stabilizing muscles, rehabilitation strategies can be optimized not only for hip OA patients but potentially for other musculoskeletal disorders affecting lower limb function. This study highlights the importance of muscle mechanics in postoperative recovery and provides a foundation for refining rehabilitation practices to improve outcomes in patients with hip OA. Further research should explore long-term muscle adaptations and their implications for functional recovery.

## 5. Conclusions

Observed reduction in tension and stiffness of the gluteus muscles prior to surgery suggests that osteoarthritis affects joint stability and forces compensatory activity from the rectus femoris and biceps femoris muscles.

The asymmetry in the rectus femoris and biceps femoris muscles between the affected and unaffected sides, both before surgery and immediately after the procedure, may indicate that these muscles play a role in the compensatory activity of the lower limbs.

Rehabilitation after hip arthroplasty is crucial for restoring muscle function, particularly in improving flexibility and reducing compensatory muscle tension.

The gluteus maximus plays a central role in stabilizing the joint after surgery, while changes in the rectus femoris are less pronounced.

The biceps femoris muscle demonstrates positive postoperative adaptation, highlighting the effectiveness of surgical and rehabilitation interventions in restoring its function.

## Figures and Tables

**Table 1:** Characteristics of the research group. Data are presented as means ± SE. Potential confounders were presented according to sex.

Variable		Research Group (*n* = 40)
Age [years]	M	63.35 ± 2.55
	F	66.18 ± 1.84
Height [cm]	M	175.43 ± 1.21 ^a^
	F	162.88 ± 1.22 ^b^
Weight [kg]	M	93.04 ± 3.05 ^a^
	F	74.47 ± 2.18 ^b^
BMI	M	29.23 ± 0.90
	F	28.48 ± 1.01
Marital status	single	-
	married	80% (32)
	widower	15% (6)
	divorced	5% (2)
Education	primary	2.5% (1)
	basic vocational	7.5% (3)
	secondary	47.5% (19)
	higher	42.5% (17)
Place of residence	village	12.5% (5)
	town	32.5% (13)
	city	55% (22)
Time since diagnosis [years]	0–1	17.5% (7)
	2–5	60% (24)
	6–10	22.5% (9)
	over 10	-
Duration of pain [years]	0–1	7.5% (3)
	2–5	57.5% (23)
	6–10	32.5% (13)
	12	-
	over 15	2.5% (1)

a and b letters indicate significantly (at *p* < 0.05) different groups measured with Student’s *t*-test.

**Table 2:** Assessment of pain level, balance, and functional ability. Data are presented as means ±SE.

Scale		Before Surgery	8–10 Days After Surgery	One Month After Surgery	Statistics
VAS		6.38 ^a^ (±0.28)	4.78 ^b^ (±0.30)	1.88 ^c^ (±0.22)	χ^2^ = 63.373 W = 0.792 *p* < 0.001
WOMAC scale		49.84 ^a^ (±2.25)	46.90 ^a^ (±1.64)	23.39 ^b^ (±1.49)	χ^2^ = 62.18 W = 0.778 *p* < 0.001
Tinetti scale	balance	10.38 ^a^ (±0.34)	7.55 ^b^ (±0.30)	10.90 ^a^ (±0.33)	χ^2^ = 34.06 W = 0.426 *p* < 0.001
	gait	8.80 ^a^ (±0.37)	7.78 ^b^ (±0.29)	9.90 ^a^ (±0.20)	χ^2^ = 34.57 W = 0.432 *p* < 0.001
	balance level	18.73 ^a^ (±0.74)	15.33 ^b^ (±0.47)	20.80 ^a^ (±0.43)	χ^2^ = 31.56 W = 0.395 *p* < 0.001

a, b, and c indicate statistically different measurements at *p* < 0.05; SE—standard error; W—Kendall’s W value (measure of effect size—0.1–0.3: small effect, 0.3–0.5: moderate effect and ≥0.5: large effect); χ^2^—Friedman χ test statistic value.

**Table 3:** Tension, stiffness, and flexibility values of the gluteus maximus muscle in both limbs at all stages of the examination. Data are presented as means ± SE.

	Before Surgery		8–10 Days After Surgery		One Month After Surgery		*p*-Value_time_ (ES)
	Not Operated Limb	Operated Limb	Not Operated Limb	Operated Limb	Not Operated Limb	Operated Limb	
Tension F (Hz)	11.63 ±0.41	11.57 ^a^ ±0.32	11.63 ±0.23	12.15 ^b^ ±0.38	10.78 ±0.13	11.10 ^a^ ±0.19	*p* = 0.086 (0.061)
							*p* = 0.009 (0.118)
*p*-value_side_	*p* = 0.969 (<0.001)		*p* = 0.363 (0.021)		*p* = 0.179 (0.046)		
Stiffness S (N/m)	224.9 ±7.8	215.1 ±5.7	215.4 ±7.7	232.2 ±12.8	216.9 ±4.8	214.7 ±3.9	*p* = 0.368 (0.025)
							*p* = 0.397 (0.023)
*p*-value_side_	*p* = 0.355 (0.021)		*p* = 0.088 (0.067)		*p* = 0.737 (0.002)		
Flexibility D (a.u.)	1.49 ±0.06	1.48 ^a^ ±0.06	1.62 ±0.08	1.61 ^a^ ±0.10	1.68 ±0.06	1.78 ^b^ ±0.08	*p* = 0.052 (0.079)
							*p* = 0.014 (0.131)
*p*-value_side_ (ES)	*p* = 0.988 (<0.001)		*p* = 0.488 (0.012)		*p* = 0.529 (0.010)		

ES—effect size (Kendall’s W value was used as a measure of effect size in repeated measurements: 0.1—small effect, 0.3—moderate effect, and ≥0.5—large effect, while for the comparisons between operated and not operated limb, η^2^ based on the Z statistics of the Mann–Whitney U test was applied: 0.01—small effect, 0.06—moderate effect, and ≥0.14—large effect). a and b indicate statistically different measurements between time points at *p* < 0.05; *p*-value_side_ indicates whether the observed differences between groups operated and not operated limb are statistically significant; *p*-value_time_ shows the differences between time points for the same limb; SE—standard error.

**Table 4:** Tension, stiffness, and flexibility values of the rectus femoris muscle in both limbs at all stages of the examination. Data are presented as means ± SE.

	Before Surgery		8–10 Days After Surgery		One Month After Surgery		*p*-Value_time_ (ES)
	Not Operated Limb	Operated Limb	Not Operated Limb	Operated Limb	Not Operated Limb	Operated Limb	
Tension F (Hz)	14.67 ±0.32	14.89 ±0.31	14.44 ±0.27	15.18 ±0.29	14.57 ±0.23	15.05 ±0.25	*p* = 0.944 (0.001)
							*p* = 0.733 (0.008)
*p*-value_side_ (ES)	*p* = 0.534 (0.009)		*p* = 0.049 (0.095)		*p* = 0.161 (0.049)		
Stiffness S (N/m)	288.2 ±8.4	296.4 ±7.1	281.40 ±6.2	294.2 ±7.2	282.6 ±4.1	288.7 ±4.9	*p* = 0.498 (0.018)
							*p* = 0.905 (0.003)
*p*-value_side_ (ES)	*p* = 0.747 (0.003)		*p* = 0.173 (0.046)		*p* = 0.292 (0.028)		
Flexibility D (a.u.)	1.58 ±0.06	1.61 ±0.06	1.52 ±0.05	1.64 ±0.07	1.63 ±0.04	1.73 ±0.06	*p* = 0.265 (0.033)
							*p* = 0.103 (0.057)
*p*-value_side_ (ES)	*p* = 0.689 (0.004)		*p* = 0.246 (0.065)		*p* = 0.225 (0.037)		

ES–effect size (the Kendall’s W value was used as a measure of effect size in repeated measurements: 0.1–small effect, 0.3–moderate effect and ≥0.5–large effect, while for the comparisons between operated and not operated limb η^2^ based on the Z statistics of Mann–Whitney U test was applied: 0.01–small effect, 0.06–moderate effect; ≥0.14–large effect). *p*-value_side_ indicates whether the observed differences between groups operated and not operated limb are statistically significant; *p*-value_time_ shows the differences between time points for the same limb; SE–standard error.

**Table 5:** Tension, stiffness, and flexibility values of the biceps femoris muscle in both limbs at all stages of the examination. Data are presented as means ± SE.

	Before Surgery		8–10 Days After Surgery		Month After Surgery		*p*-Value_time_ (ES)
	Not Operated Limb	Operated Limb	Not Operated Limb	Operated Limb	Not Operated Limb	Operated Limb	
Tension F (Hz)	13.46 ±0.41	14.63 ^a^ ±0.55	14.46 ±0.44	14.86 ^a^ ±0.55	13.99 ±0.33	13.19 ^b^ ±0.28	*p* = 0.248 (0.032)
							*p* = 0.015 (0.155)
*p*-value_side_	*p* = 0.035 (0.099)		*p* = 0.477 (0.013)		*p* = 0.049 (0.084)		
Stiffness S (N/m)	260.6 ±11.2	269.8 ^a^ ±14.6	260.3 ±10.7	269.3 ^a^ ±13.5	256.4 ±8.8	228.9 ^b^ ±7.2	*p* = 0.756 (0.008)
							*p* = 0.001 (0.182)
*p*-value_side_	*p* = 0.749 (0.003)		*p* = 0.572 (0.007)		*p* = 0.035 (0.109)		
Flexibility D (a.u.)	1.68 ±0.07	1.53 ±0.09	1.50 ±0.07	1.65 ±0.07	1.53 ±0.05	1.58 ±0.06	*p* = 0.182 (0.038)
							*p* = 0.251 (0.037)
*p*-value_side_	*p* = 0.207 (0.040)		*p* = 0.132 (0.056)		*p* = 0.724 (0.003)		

ES—effect size (Kendall’s W value was used as a measure of effect size in repeated measurements: 0.1—small effect, 0.3—moderate effect, and ≥0.5—large effect, while for the comparisons between operated and the not operated limb, η^2^ based on the Z statistics of the Mann–Whitney U test was applied: 0.01—small effect, 0.06—moderate effect, and ≥0.14—large effect). a and b indicate statistically different measurements between time points at *p* < 0.05; *p*-value_side_ indicates whether the observed differences between operated and not operated limb groups are statistically significant; *p*-value_time_ shows the differences between time points for the same limb; SE—standard error.

## Data Availability

The datasets used and/or analyzed during the current study are available from the corresponding author on reasonable request.

## Supplementary Materials

The following supporting information can be downloaded at: https://www.mdpi.com/article/10.3390/jcm14030976/s1, Table S1: Correlation coefficient between potential confounders (measured before the procedure) and tension, stiffness and flexibility of the gluteus maximus muscle in both limbs at all stages of the examination; Table S2: Correlation coefficient between potential confounders (measured before the procedure) and tension, stiffness and flexibility of the rectus femoris muscle in both limbs at all stages of the examination; Table S3: Correlation coefficient between potential confounders (measured before the procedure) and tension, stiffness and flexibility of the biceps femoris muscle in both limbs at all stages of the examination.
