# Supplementary material for: Monitoring Hip Joint Muscle Function in Osteoarthritis Patients Following Arthroplasty: A Prospective Cohort Study
Source: J Clin Med. 2025 Feb 3;14(3):976. doi: 10.3390/jcm14030976 (PMC11818077; doi:10.3390/jcm14030976)
Supplement: Supplementary file 1 [file jcm-14-00976-s001.zip › jcm-3402775-supplementary.pdf]

SUPPLEMENTARY

Table S1. Correlation coefficient between potential confounders (measured before the procedure) and tension, stiffness and flexibility of the *gluteus maximus* muscle in both limbs at all stages of the examination

Condition		Operated								
Confounder	Sex	Tension			Stiffness			Flexibility		
		Before surgery	8-10 days after surgery	Month after surgery	Before surgery	8-10 days after surgery	Month after surgery	Before surgery	8-10 days after surgery	Month after surgery
Age	M	-0,347	0,008	0,182	0,214	0,148	-0,148	0,300	0,127	-0,354
	F	0,005	0,147	0,240	-0,151	-0,036	-0,060	-0,159	0,167	-0,023
Height	M	0,311	-0,137	-0,325	-0,051	0,131	-0,175	-0,191	0,043	0,328
	F	-0,263	-0,039	-0,318	0,301	0,042	0,099	0,390	-0,253	-0,168
Weight	M	0,074	0,312	-0,386	0,052	0,373	0,004	0,086	0,138	0,047
	F	0,060	0,166	0,295	0,110	-0,355	-0,208	-0,063	0,111	-0,059
BMI	M	-0,072	0,291	-0,247	0,056	0,340	0,161	0,137	0,195	-0,036
	F	0,190	0,197	0,448	0,077	-0,306	-0,009	-0,174	0,243	0,007
VAS	M	-0,199	-0,026	-0,317	-0,275	0,055	-0,031	0,330	-0,121	0,270
	F	0,354	0,304	0,310	0,058	-0,042	-0,349	-0,160	0,031	0,186
WOMAC	M	0,067	0,192	-0,312	0,003	0,229	0,063	-0,238	-0,043	0,328
	F	-0,001	0,191	0,148	-0,237	-0,139	-0,229	-0,331	-0,077	0,472
TINETTI	M	0,122	-0,003	0,307	0,059	-0,178	0,340	0,047	-0,148	-0,020
	F	-0,312	-0,371	-0,316	0,286	0,394	0,214	0,361	-0,069	-0,349
Condition		Not operated								
Confounder	Sex	Tension			Stiffness			Flexibility		
		Before surgery	8-10 days after surgery	Month after surgery	Before surgery	8-10 days after surgery	Month after surgery	Before surgery	8-10 days after surgery	Month after surgery
Age	M	0,033	0,021	0,297	0,341	0,312	0,120	0,395	0,225	-0,226
	F	0,071	0,005	0,072	0,357	-0,015	-0,158	0,377	0,187	-0,345
Height	M	0,196	-0,012	-0,242	-0,234	-0,199	-0,367	-0,240	-0,255	0,115
	F	-0,235	0,014	-0,202	-0,174	-0,360	-0,152	-0,195	-0,226	0,063
Weight	M	-0,043	0,173	-0,053	-0,132	-0,034	0,049	0,014	-0,216	0,178
	F	0,121	0,128	0,055	-0,038	-0,008	0,143	-0,106	-0,020	0,088
BMI	M	-0,078	0,191	0,030	-0,002	0,066	0,176	0,099	-0,123	0,135
	F	0,242	0,081	0,239	0,055	0,183	0,105	0,011	0,130	0,076
VAS	M	-0,374	0,194	0,331	-0,089	0,110	0,060	0,250	-0,009	0,166
	F	0,334	0,439	0,003	0,264	-0,133	0,023	-0,332	0,291	-0,410
WOMAC	M	0,160	0,097	-0,136	-0,123	-0,272	-0,087	-0,066	-0,322	-0,098
	F	-0,023	0,102	0,197	-0,095	-0,173	0,120	0,001	0,081	-0,353
TINETTI	M	-0,043	-0,005	-0,299	0,287	0,297	0,083	-0,082	0,222	0,135
	F	-0,129	-0,386	-0,390	-0,002	0,223	-0,294	0,202	-0,190	0,234

Table S2. Correlation coefficient between potential confounders (measured before the procedure) and tension, stiffness and flexibility of the *rectus femoris* muscle in both limbs at all stages of the examination

Condition		Operated								
Confounder	Sex	Tension			Stiffness			Flexibility		
		Before surgery	8-10 days after surgery	Month after surgery	Before surgery	8-10 days after surgery	Month after surgery	Before surgery	8-10 days after surgery	Month after surgery
Age	M	0,340	0,129	0,060	0,222	0,007	0,036	0,209	0,002	-0,077
	F	-0,043	0,173	-0,053	-0,132	-0,034	0,049	0,014	-0,216	0,178
Height	M	-0,272	-0,100	0,139	-0,057	-0,213	-0,209	-0,062	-0,292	-0,100
	F	-0,078	0,191	0,030	-0,002	0,066	0,176	0,099	-0,123	0,135
Weight	M	-0,204	0,164	-0,375	-0,346	-0,054	-0,273	0,185	-0,098	-0,227
	F	-0,030	0,239	0,376	-0,459	-0,407	-0,013	0,039	-0,135	0,430
BMI	M	-0,163	0,126	-0,381	-0,259	-0,095	-0,241	0,362	0,024	-0,230
	F	0,075	0,123	0,239	-0,359	-0,369	-0,195	0,088	-0,030	0,411
VAS	M	0,345	0,238	-0,285	0,199	-0,236	-0,278	-0,085	-0,235	0,043
	F	0,267	-0,394	0,231	0,235	-0,067	-0,223	0,033	0,101	0,123
WOMAC	M	0,387	-0,035	-0,210	0,412	0,218	-0,257	0,080	0,238	0,382
	F	0,358	-0,138	0,291	0,187	-0,084	-0,023	-0,053	0,180	0,006
TINETTI	M	-0,251	-0,299	0,402	-0,317	-0,289	0,256	-0,061	-0,022	0,067
	F	-0,360	0,149	-0,233	-0,067	0,092	0,164	0,084	-0,371	0,084
Condition		Not operated								
Confounder	Sex	Tension			Stiffness			Flexibility		
		Before surgery	8-10 days after surgery	Month after surgery	Before surgery	8-10 days after surgery	Month after surgery	Before surgery	8-10 days after surgery	Month after surgery
Age	M	0,203	0,337	0,131	0,366	0,389	-0,041	-0,156	0,071	-0,066
	F	-0,274	-0,128	0,241	-0,102	0,229	-0,200	-0,027	0,395	-0,119
Height	M	0,196	-0,012	-0,242	-0,234	-0,199	-0,367	-0,240	-0,255	0,115
	F	-0,235	0,014	-0,202	-0,174	-0,360	-0,152	-0,195	-0,226	0,063
Weight	M	-0,175	-0,130	-0,054	-0,199	-0,340	-0,230	-0,051	-0,291	-0,155
	F	-0,104	-0,414	-0,039	-0,380	-0,480	-0,320	-0,383	-0,290	-0,125
BMI	M	-0,136	-0,124	0,065	-0,115	-0,410	-0,305	0,017	-0,260	-0,306
	F	-0,084	-0,359	0,026	-0,300	-0,280	-0,315	-0,227	-0,058	-0,119
VAS	M	-0,097	0,175	-0,119	0,033	0,120	-0,289	0,031	-0,044	0,219
	F	0,281	0,229	0,434	0,411	0,103	0,258	0,264	0,371	0,304
WOMAC	M	0,193	0,056	0,309	0,305	0,033	0,268	0,389	0,228	0,341
	F	0,334	0,219	0,302	0,110	0,065	0,371	0,239	0,210	0,290
TINETTI	M	-0,035	0,181	-0,169	-0,080	0,034	0,155	-0,174	-0,145	0,245
	F	0,034	0,023	-0,102	0,012	-0,153	0,143	0,198	-0,242	-0,097

Table S3. Correlation coefficient between potential confounders (measured before the procedure) and tension, stiffness and flexibility of the *biceps femoris* muscle in both limbs at all stages of the examination

Condition		Operated								
Confounder	Sex	Tension			Stiffness			Flexibility		
		Before surgery	8-10 days after surgery	Month after surgery	Before surgery	8-10 days after surgery	Month after surgery	Before surgery	8-10 days after surgery	Month after surgery
Age	M	0,220	0,143	0,160	0,112	0,052	0,036	0,209	0,002	-0,077
	F	-0,125	0,009	0,122	0,058	0,169	-0,254	0,158	0,388	0,403
Height	M	-0,277	-0,100	0,139	-0,057	-0,213	-0,209	-0,062	-0,292	-0,100
	F	0,004	0,067	0,071	0,344	-0,011	-0,015	-0,076	0,004	0,067
Weight	M	-0,204	0,164	-0,235	-0,346	-0,054	-0,273	0,185	-0,098	-0,227
	F	-0,030	0,239	0,376	-0,459	-0,407	-0,013	0,039	-0,135	0,430
BMI	M	-0,163	0,126	-0,381	-0,259	-0,095	-0,241	0,362	0,024	-0,230
	F	0,075	0,123	0,239	-0,359	-0,369	-0,195	0,088	-0,030	0,411
VAS	M	0,345	0,238	-0,285	0,199	-0,236	-0,278	-0,085	-0,235	0,043
	F	0,267	-0,394	0,231	0,235	-0,067	-0,223	0,033	0,101	0,123
WOMAC	M	0,387	-0,035	-0,210	0,412	0,218	-0,257	0,080	0,238	0,382
	F	0,358	-0,138	0,291	0,187	-0,084	-0,023	-0,053	0,180	0,006
TINETTI	M	-0,251	-0,299	0,402	-0,317	-0,289	0,256	-0,061	-0,022	0,067
	F	-0,360	0,149	-0,233	-0,067	0,092	0,164	0,084	-0,371	0,084

Condition		Not operated								
Confounder	Sex	Tension			Stiffness			Flexibility		
		Before surgery	8-10 days after surgery	Month after surgery	Before surgery	8-10 days after surgery	Month after surgery	Before surgery	8-10 days after surgery	Month after surgery
Age	M	0,203	0,337	0,131	0,366	0,389	-0,041	-0,156	0,071	-0,066
	F	-0,274	-0,128	0,241	-0,102	0,229	-0,200	-0,027	0,395	-0,119
Height	M	0,196	-0,012	-0,242	-0,234	-0,199	-0,367	-0,240	-0,255	0,115
	F	-0,235	0,014	-0,202	-0,174	-0,360	-0,152	-0,195	-0,226	0,063
Weight	M	-0,175	-0,130	-0,054	-0,199	-0,340	-0,230	-0,051	-0,291	-0,155
	F	-0,104	-0,414	-0,039	-0,380	-0,480	-0,320	-0,383	-0,290	-0,125
BMI	M	-0,136	-0,124	0,065	-0,115	-0,410	-0,305	0,017	-0,260	-0,306
	F	-0,084	-0,359	0,026	-0,300	-0,280	-0,315	-0,227	-0,058	-0,119
VAS	M	-0,097	0,175	-0,119	0,033	0,120	-0,289	0,031	-0,044	0,219
	F	0,281	0,229	0,434	0,411	0,103	0,258	0,264	0,371	0,304
WOMAC	M	0,193	0,056	0,309	0,305	0,033	0,268	0,389	0,228	0,341
	F	0,334	0,219	0,302	0,110	0,065	0,371	0,239	0,210	0,290
TINETTI	M	-0,035	0,181	-0,169	-0,080	0,034	0,155	-0,174	-0,145	0,245
	F	0,034	0,023	-0,102	0,012	-0,153	0,143	0,198	-0,242	-0,097
